# Additional surgical procedures and perioperative morbidity in post-chemotherapy retroperitoneal lymph node dissection for metastatic testicular cancer in two intermediate volume hospitals

**DOI:** 10.1007/s00345-020-03229-5

**Published:** 2020-05-05

**Authors:** Joost M. Blok, Richard P. Meijer, Henk G. van der Poel, Axel Bex, Jeanette van Vooren, Japke J. van Urk, Simon Horenblas, J. L. H. Ruud Bosch

**Affiliations:** 1 Department of Oncological Urology, University Medical Center Utrecht, Utrecht, The Netherlands; 2 Department of Urology, The Netherlands Cancer Institute, Amsterdam, The Netherlands; 3 Department of Radiology, University Medical Center Utrecht, Utrecht, The Netherlands; 4 Department of Radiology, The Netherlands Cancer Institute, Amsterdam, The Netherlands

**Keywords:** Testicular cancer, Testicular germ cell tumor, Retroperitoneal lymph node dissection, Morbidity

## Abstract

**Purpose:**

To evaluate the perioperative morbidity of PC-RPLND in two intermediate volume centers and to identify predictors of high morbidity.

**Methods:**

Retrospective analysis of 124 patients treated with open PC-RPLND at two tertiary referral centers between 2001 and 2018. Perioperative morbidity was determined by analyzing additional surgical procedures, intra-operative blood loss, and postoperative complications.

**Results:**

An additional procedure was necessary for 33 patients (26.6%). The risk was higher in patients with IGCCCG intermediate/poor prognosis (OR 3.56; 95% CI 1.33–9.52) and residual tumor size > 5 cm (OR 3.53; 95% CI 1.39–8.93). Blood loss was higher in patients with IGCCCG intermediate/poor prognosis (*β =* 0.177; *p =* 0.029), large residual tumor (*β =* 0.570; *p* < 0.001), an additional intervention (*β =* 0.342; *p* < 0.001) and teratoma on retroperitoneal histology (*β =* − 0.19; *p =* 0.014). Thirty-one patients had a postoperative complication Clavien-Dindo Grade ≥ 2 (25.0%). Complication risk was highest in patients undergoing an additional intervention (OR 3.46; 95% CI 1.03–11.60; *p =* 0.044).

**Conclusions:**

The rate of additional interventions in our series is comparable to what has been reported in high-volume centers. IGCCCG intermediate/poor prognosis patients with high-volume disease and patients undergoing an additional surgical procedure can be classified as high-risk patients.

**Electronic supplementary material:**

The online version of this article (10.1007/s00345-020-03229-5) contains supplementary material, which is available to authorized users.

## Introduction

Post-chemotherapy retroperitoneal lymph node dissection (PC-RPLND) is an important component of the treatment of disseminated germ cell tumor (GCT) [1–4]. It is a technically challenging procedure and is associated with significant treatment-related morbidity [5, 6]. In up to 30% of procedures, an additional surgical intervention is necessary during the procedure (e.g. nephrectomy or vascular reconstruction) [5, 7–9]. However, the identification of patients that are at increased risk of an additional procedure is primarily based on preoperative imaging.

Previous publications about the outcome of RPLND are mainly from high volume centers and these reports make the case for further centralization [10–12]. It is debatable whether these large series reflect the outcome of the procedure in general. After all, most patients are not treated in one of the leading centers of the world. For example, the median annual number of RPLNDs per urologist in the USA is only one [13]. Between 2003 and 2013, 75% of urologists performed just one RPLND, while three urologists logged 23% of all procedures. These findings are confirmed by Yu et al., who showed that 51.6% of RPLNDs in the USA were performed at hospitals with ≤ 2 procedures annually [14]. In their analysis of German hospital billing data covering 2006–2015, Groeben et al. found that 44% of RPLNDs were performed in a low volume center (< 4 cases annually) [15]. Although there was a modest trend towards centralization, still only 18% of all RPLNDs in 2015 were performed in a high volume institution (> 10 cases annually). Thus, although most publications about PC-RPLND concern the outcomes in high volume centers, the overall majority of patients are treated in a low volume center.

In smaller countries, such as The Netherlands, the low incidence of testicular cancer prevents the establishment of very high volume centers. Since 2017, the quality standards of the Dutch urological society state that a center offering RPLND should perform at least ten procedures annually [16].

Although it has been shown that the overall complication risk of RPLND is significantly lower in hospitals with a higher volume [14], reports from low and intermediate volume centers are still scarce. These reports are important to give a true view of the morbidity of PC-RPLND.

In the present study, we evaluate the perioperative morbidity of PC-RPLND in two intermediate volume centers. Our primary aim is to analyze whether the perioperative morbidity is comparable to what has been reported in the literature. Our secondary aim is to investigate whether there are any risk factors that can be used to better identify patients with a high risk of perioperative morbidity.

## Materials and methods

We performed a retrospective analysis of the medical records of all patients who were treated with open PC-RPLND in two tertiary referral centers between 2001 and 2018. In both centers, surgery was indicated in case of retroperitoneal residual tumor > 1 cm after at least three cycles of cisplatin-based combination chemotherapy (bleomycin, etosposide, cisplatin). All patients who were treated with open PC-RPLND for gonadal or extragonadal GCT between 2001 and 2018 were included in our analysis. Exclusion criteria were incomplete data, prior retroperitoneal radiotherapy, prior RPLND (re-do RPLND), elevated tumor markers at time of surgery (desperation RPLND) and a minimally-invasive procedure. Patients who were previously treated with salvage chemotherapy but had normal tumor markers at the time of surgery were also eligible for inclusion. Institutional review board approval was obtained from both centers.

During the period covered by our analysis, patients with a small tumor (< 5 cm) that was not adjacent to the large vessels were mainly treated with a minimally-invasive procedure at the Netherlands Cancer Institute (NCI). These patients were excluded from the present analysis.

All patients at the University Medical Center Utrecht (UMCU) underwent a template-based RPLND. In case of a residual tumor < 5 cm in the primary landing zone, a modified template was applied. In right-sided modified template dissection, the right ureter and the aorta were the lateral and medial boundaries, respectively. The renal vein was the cranial boundary and the crossing of the ureter over the common iliac vessels was the caudal boundary. In left-sided dissection, the lateral, cranial and caudal boundaries were represented by the ureter, the renal vein, and the crossing of the ureter over the common iliac vessels, respectively.

At the NCI, complete removal of the residual mass and all enlarged lymph nodes identified on imaging and during surgery were resected, but no template resection of clinically and radiologically unsuspicious lymph nodes was done. The tumor localization prior to chemotherapy was taken into account.

An additional procedure was defined as any surgical intervention that was performed in the same surgical session as the PC-RPLND.

Complications that occurred during the 30-day postoperative period were categorized according to the Clavien-Dindo classification of surgical complications [17]. In the case of multiple complications in one patient, all complications were registered but only the highest grade was used for the statistical analysis of risk factors.

The available abdominal computed tomography (CT) scans prior to chemotherapy and prior to surgery were re-analyzed by one of two independent radiologists (J.V. and J.U.). They measured the tumor mass in three dimensions (axial, coronal, and sagittal) and examined whether the additional interventions could be predicted on the basis of these scans.

Variables significant at the *p* < 0.10 level in the univariate logistic regression analysis were considered for inclusion in the multivariate logistic regression analysis. Multiple regression analysis was performed to analyze the association between intra-operative blood loss as a continuous variable and relevant predictor variables. We corrected for the type of surgery (template-based RPLND vs. residual mass resection [RMR]) and primary histology. All tests were two-tailed and *p* value < 0.05 was considered significant. SPSS version 22 (IBM Corp., USA) was used for statistical analysis.

## Results

A total of 148 open PC-RPLNDs were identified between 2001 and 2018. Twenty-four patients were excluded because of a history of prior RPLND (*n =* 11), elevated tumor markers (*n =* 10), missing operative report (*n =* 2), or history of retroperitoneal radiotherapy (*n =* 1). The remaining 124 patients (seminoma *n =* 17; nonseminomatous germ cell tumor [NSGCT] *n =* 107) were included in the present analysis (Table 1).

**Table 1:** Patient characteristics and operative outcome

	Overall	Template RPLND	RMR	*p* value
Patients, no	124	72	52	
Median age at surgery, years (IQR)	29.8 (24.4–37.5)	28.5 (24.4–35.0)	32.3 (24.5–40.1)	0.104
Retroperitoneal primary, no. (%)	14 (11.3)	9 (12.5)	5 (9.6)	0.776
Histologic subtype primary tumor, no. (%)				0.792
Non-seminoma	107 (86.3)	63 (87.5)	44 (84.6)	
Seminoma	17 (13.7)	9 (12.5)	8 (15.4)	
IGCCCG risk classification, no. (%)				0.120
Good	56 (42.2)	37 (51.4)	19 (36.5)	
Intermediate	43 (34.7)	24 (33.3)	19 (36.5)	
Poor	24 (19.4)	10 (13.9)	14 (26.9)	
Missing	1 (0.8)	1 (1.4)	0	
Median diameter residual tumor, cm (IQR)	4.7 (2.9–8.0)	3.9 (2.4–6.9)	6.1 (3.9–8.8)	0.010
≤ 5 cm, no. (%)	67 (54.0)	46 (63.9)	21 (40.4)	
> 5–10 cm, no. (%)	38 (30.6)	16 (22.2)	22 (42.3)	
> 10 cm, no. (%)	19 (15.3)	10 (13.9)	9 (17.3)	
Median operative time, mins (IQR)	248 (178–343)	275 (202–356)	217 (139–330)	0.009
With additional procedure	360 (264–433)	409 (350–465)	280 (141–362)	
Without additional procedure	233 (173–297)	245 (193–300)	184 (135–289)	
Median blood loss, ml (IQR)	890 (400–2080)	500 (250–1372)	1265 (570–3000)	0.001
With additional procedure	2008 (800–3315)	1800 (1050–3500)	2015 (650–3230)	
Without additional procedure	700 (325–1505)	400 (190–600)	1100 (535–2143)	
Additional surgical procedures, pts. (%)	33 (26.6)	16 (22.2)	17 (32.7)	0.220
Nephrectomy	9 (7.3)	6 (8.3)	3 (5.8)	0.733
IVC resection/reconstruction	8 (6.5)	2 (2.8)	6 (11.5)	0.068
Aorta reconstruction	6 (4.8)	4 (5.6)	2 (3.8)	1.00
Iliac artery reconstruction	7 (5.6)	2 (2.8)	5 (9.6)	0.129
Renal vein reconstruction	5 (4.0)	4 (5.6)	1 (1.9)	0.398
Median postoperative stay, days (IQR)	7 (5–9)	7 (6–9)	7 (5–8)	0.084
Patients with postoperative complications ≥ Grade 2 (%)	31 (25.0)	19 (26.4)	12 (23.1)	0.834
Clavien-Dindo Grade 2	24 (19.4)	12 (16.7)	12 (23.1)	0.812
Clavien-Dindo Grade 3a	4 (3.2)	3 (4.2)	1 (1.9)	0.641
Clavien-Dindo Grade 3b	5 (4.0)	4 (5.6)	1 (1.9)	0.402
Clavien-Dindo Grade 4a	3 (2.4)	2 (2.8)	1 (1.9)	1.00
Clavien-Dindo Grade 5	2 (1.6)	1 (1.4)	1 (1.9)	1.00
Histology lymphadenectomy specimen, no. (%)				0.936
Teratoma	62 (50.0)	36 (50.0)	26 (50.0)	
Fibrosis/necrosis	46 (37.1)	26 (36.1)	20 (38.5)	
Viable cancer	16 (12.9)	10 (13.9)	6 (11.5)	

*IGCCCG* International Germ Cell Cancer Group, *IVC* inferior vena cava, *IQR* interquartile range, *RMR* residual mass resection, *RPLND* retroperitoneal lymph node dissection

Eleven surgeons performed at least one of the procedures. Five surgeons had a volume of more than ten procedures and performed a combined total of 106 procedures. The remaining 16 procedures were divided among 6 surgeons.

Seventy-two patients were treated with template-based surgery and 52 patients with residual mass resection. Fifteen patients (12.1%) had received salvage chemotherapy prior to surgery. The median residual tumor size was larger in the RMR group (6.1 cm), compared to the RPLND group (3.9 cm; *p =* 0.010). Patients in the RMR group had more often International Germ Cell Cancer Collaborative Group (IGCCCG) intermediate/poor prognosis (63.5%), compared to patients in the RPLND group (47.2%).

A total of 33 patients (26.6%) required 46 additional surgical procedures (Table 1). Most common interventions were nephrectomy (*n =* 9; 7.3%) and inferior vena cava (IVC) resection/reconstruction (*n =* 8; 6.5%). Less common interventions were: partial bowel resection, renal artery resection (each *n =* 3; 2.4%), partial liver resection (*n =* 2; 1.6%), adrenalectomy, superior mesenteric artery reconstruction, and segmental ureter resection with ureteroureterostomy (each *n =* 1, 0.8%). Assistance of a vascular surgeon was required in 20 cases (16.1%). An additional procedure was performed in 16/72 patients undergoing template RPLND (22.2%) and 17/52 patients undergoing residual mass resection (32.7%).

In all, 29 of 46 additional interventions (63.0%) were performed to achieve an adequate resection and 17 interventions (37.0%) were the consequence of an intraoperative complication. These complications were lesions of the iliac artery (*n =* 6), aorta (*n =* 4), renal artery (*n =* 3), renal vein (*n =* 2), IVC (*n =* 1) and superior mesenteric artery (*n =* 1). The tumor was adjacent to the site of additional intervention in all cases, which suggests that a preoperative CT scan is sufficient to identify patients in whom an additional intervention is likely to be necessary.

The necessity of an additional surgical procedure was significantly associated with IGCCCG intermediate/poor prognosis and residual tumor size > 5 cm (Table 2). Pure seminoma on primary histology and type of surgery were not significantly associated with an additional intervention. On multivariate analysis, intermediate/poor risk category (OR 3.56; 95% CI 1.33–9.52; *p =* 0.011) and tumor size > 5 cm (OR 3.53; 95% CI 1.39–8.93; *p =* 0.008) were significant predictors of an additional intervention. Taking only the 107 patients with NSGCT into account, tumor size > 5 cm was still a significant predictor (OR 3.38; 95% CI 1.23–9.27; *p =* 0.018) but intermediate/poor prognosis became borderline insignificant (OR 2.72; 95% CI 0.93–7.97; *p =* 0.068; Supplementary Table 1).

**Table 2:** Predictors of additional surgical procedures

	Univariate		Multivariate	
	OR (95% CI)	*p* value	OR (95% CI)	*p* value
Age	0.99 (0.95–1.03)	0.640		
Left-sided primary	0.67 (2.88–1.58)	0.361		
Retroperitoneal primary	1.63 (0.50–5.27)	0.426		
Seminoma primary	1.62 (0.55–4.79)	0.395	1.47 (0.46–4.75)	0.521
IGCCCG intermediate/poor prognosis	**4.44 (1.75–11.27)**	**0.001**	**3.56 (1.33–9.52)**	**0.011**
Tumor regression	1.01 (0.99–1.04)	0.188		
Residual tumor size > 5 cm	**4.69 (1.95–11.27)**	** < 0.001**	**3.53 (1.39–8.93)**	**0.008**
Residual mass resection^a^	1.70 (0.76–3.79)	0.195	1.09 (0.45–2.66)	0.852
Histology RPLND specimen		0.761		
Necrosis/fibrosis	Reference			
Viable cancer	0.76 (0.21–2.78)	0.680		
Teratoma	0.73 (0.31–1.72)	0.470		

Significant values in bold

*IGCCCG* International Germ Cell Cancer Group, *OR* odds ratio, *RMR* residual mass resection, *RPLND* retroperitoneal lymph node dissection

^a^Compared to template-based surgery

Multiple regression analysis found that tumor regression and viable cancer on retroperitoneal histology were not significantly correlated with blood loss. Retroperitoneal primary, type of surgery, teratoma on retroperitoneal histology, additional intervention, IGCCCG prognosis and residual tumor size were included in the model. IGCCCG intermediate/poor prognosis (*β =* 0.177; *p =* 0.029), residual tumor size (*β =* 0.570; *p* < 0.001), necessity of an additional intervention (*β =* 0.342; *p* < 0.001) and teratoma on retroperitoneal histology (*β =* − 0.190; *p =* 0.014) were significantly correlated with blood loss (adjusted *R*^2^ = 0.438; *p* < 0.001).

A total of 38 postoperative complications Clavien-Dindo Grade ≥ 2 were identified in 31 patients (25.0%; Supplementary Table 2). A reoperation (Grade 3b) was necessary in three patients (3.2%). One patient underwent a hemicolectomy for colon ischemia. Another patient had a perforation of the small intestine, which was repaired during explorative laparotomy. The third patient had metabolic instability with unknown cause for which he underwent explorative laparotomy without an additional intraoperative intervention.

The risk of a severe complication (Grade ≥ 3) was higher in patients with an additional intervention (24.2%) compared to patients without an additional intervention (6.6%, *p =* 0.011) and this was borderline significant when corrected for residual tumor size (OR 3.46; 95% CI 1.03–11.60; *p =* 0.044; Supplementary Table 3). Tumor regression was not associated with an additional intervention or postoperative complication.

Two patients (1.6%) died from a postoperative complication (Grade 5). One patient had IGCCCG poor prognosis and a 10 cm large residual tumor in the left para-aortal region. The day after surgery, he developed hematochezia but exploratory laparotomy showed no signs of intestinal ischemia. A week later, the patient became hemodynamically unstable and a bleeding of the left renal artery was diagnosed, which was sutured during a subsequent surgical procedure. Unfortunately, the patient developed necrotizing pancreatitis with abdominal bleeding of unknown origin and had to undergo seven more exploratory laparotomies with resection of necrotic tissue. One month after PC-RPLND, a new aortic bleeding developed, for which an endovascular stent was placed by a vascular surgeon. Twenty-three days later, however, the patient became hemodynamically unstable again and CT-imaging showed an aortic bleeding proximally to the stent. There were no more therapeutic options and the patient died the same day.

The second patient had intermediate prognosis and a 25 cm large residual tumor. He had persistent chylous ascites for which he underwent multiple abdominal drainages. Forty-six days after surgery, a peritoneovenous shunt was placed. After 3 months, the leaking lymph vessels were ligated during laparotomy. During this procedure, the aorta had to be reconstructed by a vascular surgeon because of an intraoperative avulsion. After surgery, he developed an aortic bleeding of which he died.

After a median follow-up of 60.2 months (IQR 28.0–93.8), 9 patients (7.3%) had disease recurrence or progression. This was a retroperitoneal relapse in five patients (4.0%; template 2/72 patients, RMR 3/52 patients). All retroperitoneal relapses were inside the surgical field, except for one patient in the RMR group. The patients with retroperitoneal relapse were treated with salvage chemotherapy (*n =* 1), palliative chemotherapy (*n =* 1), chemotherapy with radiotherapy (*n =* 1) or surgery (*n =* 2). The four patients with relapse outside the retroperitoneum were all treated with chemotherapy. One patient also received radiotherapy and another patient underwent pelvic node resection in addition to his chemotherapeutic treatment.

Five patients (4.0%) died of the disease. Four of these had a retroperitoneal relapse and one had tumor recurrence in the peritoneum. The cause of death was unknown in two patients. Together with the two patients who died of a postoperative complication, nine patients in our cohort died. Follow-up was < 12 months in 14 patients. Among the remaining 110 patients, overall survival was 91.8% (template 93.9%; RMR 88.6%) and cancer-specific survival was 93.6% (template 95.5%; RMR 90.9%).

## Discussion

The rate of an additional intervention in our study is comparable to what has been reported in other series, which ranges between 13 and 38% [11, 12, 18–21]. As in our study, nephrectomy and IVC interventions are the most commonly performed additional procedures [7, 11, 12, 18–20]. Cary et al. reported the results of 755 patients of the Indiana University, which is one of the largest series to date [11]. From 2003 to 2011, the annual rate of additional procedures ranged between 17 and 30%. A nephrectomy was necessary in 7.3% of patients. In a series of 85 patients who were treated by a single surgeon between 2004 and 2010, 28 patients (33%) required adjuvant surgery [19]. This was a vascular procedure in 13 patients (15%) and a nephrectomy in 12 patients (14%). In a multicenter analysis of 339 PC-RPLNDs by the German Testicular Cancer Study Group, the rates of IVC intervention and nephrectomy were 10% and 9%, respectively [20].

The results from these institutional series are similar to what has been found in nationwide studies. Wells et al. evaluated audit data for all RPLNDs in the UK between March 2012 and February 2013 and found that the rates of synchronous nephrectomy and vascular reconstruction were 11.1% and 5%, respectively [22]. Not all RPLNDs were in the post-chemotherapy setting (72.2%), but only 5.6% of procedures were primary RPLNDs. Macleod et al. analyzed the insurance data of 206 patients undergoing PC-RPLND in the USA [23]. Overall, 19% of patients underwent an adjunctive procedure, of which nephrectomy (10%) and vascular reconstruction (8%) were the most common interventions. Thus, the rate of an additional intervention in our series is similar to what has been reported by large institutional series and nationwide cohort studies.

Postoperative complication rates reported in the literature vary widely and are primarily based on single-center series. Several high volume centers have reported rates between 3 and 12% [6, 11, 12, 18, 24]. However, several population-based studies have found much higher complication rates than what has been reported in series from high volume institutions. The study by Wells et al. showed that in only 73.5% of all RPLNDs in the UK no complication was recorded [22]. In a nationwide sample of all RPLNDs in the USA between 2001 and 2008, the overall rate of complication was 24.8% [14]. According to a population-based analysis of all PC-RPLNDs in Norway and Sweden between 2007 and 2014, a complication occurred in 25% of patients treated with unilateral PC-RPLND and 45% of bilateral PC-RPLND [25]. A Clavien-Dindo Grade ≥ 3b complication occurred after 2.2% of unilateral procedures and 9.2% of bilateral procedures. This shows that the complication rate in our cohort is equal to what has been reported in nationwide cohort studies.

Although based on only two cases, the rate of Grade 5 complications in our cohort (1.6%) is higher than what has been reported in comparative studies. In a series of 152 patients by Heidenreich et al., one patient (0.7%) died due to massive postoperative bleeding caused by an aorto-dueodenal fistula [18]. Fléchon et al. reported one death due to an intra-abdominal bleeding in a cohort of 151 patients (0.7%) [3].

Patient outcome after complex cancer surgery is correlated with hospital volume [26, 27]. For testicular malignancies specifically, the recent literature is scarce. Woldu et al. found an association between hospital volume and survival in patients with non-localized NSGCT [28]. The authors analyzed data from the National Cancer Database (USA) for patients treated for testicular germ cell tumor (TGCT) in the years 2004–2014. Compared to the highest volume hospitals, the hazard ratios for overall mortality were 1.28, 1.45, 1.48, and 1.83 for high-intermediate, intermediate, low-intermediate, and low volume hospitals, respectively. For RPLND specifically, Yu et al. showed that the overall complication risk was significantly lower in hospitals with a higher volume [14]. This shows that the centralization of RPLND is important to improve patient outcome. Although the most optimal annual number of procedures has yet to be determined, the current cutoff value of ten procedures per year in The Netherlands is relatively low.

Several reports have shown a strong association between residual tumor size and additional interventions, similar to our findings [7, 11, 20]. In the series by Cary et al., residual tumor size > 10 cm was the strongest predictor of an additional procedure (OR 7.2; 95% CI 2.6–19.5) [11]. In an earlier study by the same authors, 31.9% of patients with a residual tumor size > 10 cm had to undergo nephrectomy [7]. A recent study from the University Hospital of Dusseldorf found a higher rate of additional interventions in patients undergoing a bilateral PC-RPLND (43%), compared to a unilateral PC-RPLND (23%; *p =* 0.006) [9]. Nephrectomy was indicated in 12% of bilateral procedures but only in 3% of unilateral procedures (*p =* 0.03). This difference can be most likely attributed to the difference in tumor size, since the decision whether to perform a unilateral or bilateral procedure was based on the size and location of the residual tumor, with 5 cm as a cut-off value [9].

The correlation between IGCCCG intermediate/poor prognosis and an additional intervention has been described previously by Winter et al. [20]. The authors found that the probability of an IVC intervention increased with tumor size ≥ 5 cm and worse IGCCCG risk category. Our study shows that these risk factors also apply to non-vascular additional procedures. The association between pre-chemotherapy risk category and additional (vascular) procedures can be explained by the fact that IGCCCG prognosis group can be regarded as a measure of tumor burden. Another possibility is a more severe desmoplastic reaction in patients treated with more cycles of chemotherapy.

In addition to these patient and tumor characteristics, the indication of an additional intervention is also dependent on the PC-RPLND setting. The risk of an additional procedure is higher in patients who were treated with salvage chemotherapy [29]. Since only 15/124 patients in our cohort were treated with salvage chemotherapy and this parameter was highly correlated with IGCCCG prognosis, we did not include this parameter in our analysis.

Whether complete resection of all residual tumor outside the retroperitoneal nodes is always indicated is up for debate. Recent studies have shown that a more extensive resection does not always lead to a better outcome. Nini et al. reported on a series of 14 patients with nodal and bone involvement undergoing PC-RPLND with simultaneous partial or complete bone resection [30]. All four patients with vital cancer had disease progression, irrespective of the extent of the bone resection, and three out of four died. Among the six patients with teratoma, both patients that were treated with partial bone resection had disease progression and died, whereas the four treated with a complete resection have been cured. This suggests that a more extensive bone resection was only beneficial in patients with teratoma but not in patients with vital cancer [30]. This is in line with a study by Nestler et al., who analyzed the tumor histology in resected organs in a cohort of 235 patients undergoing PC-RPLND with an additional resection [31]. Most common interventions were nephrectomy (*n =* 74), IVC resection (*n =* 66) and partial liver resection (*n =* 48). Histopathological analysis of the resected organs showed necrosis in 40% of patients, which implies that the additional resection was oncologically unnecessary in these cases.

We have identified clinical predictors that are useful for the risk classification of PC-RPLND patients. Patients with intermediate or poor prognosis, high volume disease, or patients undergoing an additional surgical procedure can be classified as high-risk patients. Although a complete diagnostic workup is necessary for all patients, extra attention is warranted in high risk patients. Evaluation of possible tumor ingrowth in adjacent organs is of particular importance in these patients. All tumors were adjacent to the site of additional intervention in our series. This shows that a preoperative CT scan is sufficient to identify patients in which an additional intervention is necessary.

Our study is subject to certain limitations. First, a substantial portion of patients was treated with RMR instead of template-based RPLND. RMR is not standard of care and may be associated with a higher risk of retroperitoneal relapse. Although we corrected for the type of surgery in our analysis, this makes our results less generalizable. Second, its retrospective nature can lead to bias and underreporting of perioperative morbidity. We believe that the underreporting of complications is low, as we only included complications Grade ≥ 2, which are generally well reported. Third, patients at the NCI who had small volume residual disease (< 5 cm) were not included in this study, since they were treated with a minimally-invasive procedure. This may have introduced selection bias and overestimated the relapse rate, mortality rate and rate of additional interventions and complications. It also prevents a solid comparison between both surgical approaches, since the patient cohorts differed significantly. Fourth, PC-RPLND is performed at a lower frequency in our centers, compared to other larger series. Both centers, however, are two of the largest centers for PC-RPLND in The Netherlands. In addition to the treatment of low volume disease with a minimally-invasive procedure, the low frequency of this procedure can be explained by the low number of TGCT patients in our country (~ 800 new TGCT patients annually). Nevertheless, the outcomes of this study could spur the discussion on further centralization of PC-RPLND.

A key strength of our study is that a radiologist re-analyzed the CT scans prior to chemotherapy and surgery. This assured uniformity in the method of tumor measurement and calculation of tumor regression. Another strength was the long median follow-up (> 5 years) since almost all patients had their post-surgery follow-up at one of the participating centers.

## Conclusions

In conclusion, the rate of additional interventions and postoperative complications in our series is comparable to what has been reported in other reports. IGCCCG intermediate/poor prognosis patients with high-volume disease can be classified as high-risk patients. To optimize outcome, extra attention to possible tumor ingrowth and precautionary measures (e.g. assistance from a vascular surgeon, postoperative stay at the intensive care unit) is warranted in these patients. The preoperative CT scan is sufficient to identify patients in which an additional intervention is necessary.

## Electronic supplementary material

Below is the link to the electronic supplementary material.

Supplementary file1 (DOCX 19 kb)

Supplementary file2 (DOCX 18 kb)

Supplementary file3 (DOCX 16 kb)
