# Supplementary material for: Additional surgical procedures and perioperative morbidity in post-chemotherapy retroperitoneal lymph node dissection for metastatic testicular cancer in two intermediate volume hospitals
Source: World J Urol. 2020 May 5;39(3):839–46. doi: 10.1007/s00345-020-03229-5 (PMC7969692; doi:10.1007/s00345-020-03229-5)
Supplement: Supplementary file 1 — Supplementary file1 (DOCX 19 kb) [file 345_2020_3229_MOESM1_ESM.docx]

**Supplementary Table 1. Predictors of Additional Surgical Procedures in NSGCT Patients**

|  | **Univariate** | | **Multivariate** | |
| --- | --- | --- | --- | --- |
|  | **OR (95% CI)** | **P-value** | **OR (95% CI)** | **P-value** |
| Age | 0.99 (0.94-1.04) | 0.750 |  |  |
| Left-sided primary | 0.68 (0.27-1.72) | 0.409 |  |  |
| Retroperitoneal primary | 1.81 (0.49-6.75) | 0.386 |  |  |
| IGCCCG intermediate / poor risk | **3.97 (1.45-10.90)** | **0.004** | 2.72 (0.93-7.97) | 0.068 |
| Tumor regression | 1.01 (0.99-1.03) | 0.333 |  |  |
| Residual tumor size >5 cm | **4.66 (1.81-12.02)** | **0.001** | **3.38 (1.23-9.27)** | **0.018** |
| Residual mass resection* | 1.80 (0.74-4.33) | 0.192 | 1.14 (0.43-3.01) | 0.797 |
| Histology RPLND specimen |  | 0.874 |  |  |
| - Necrosis / fibrosis | Reference |  |  |  |
| - Viable cancer | 1.07 (0.27-4.28) | 0.927 |  |  |
| - Teratoma | 0.81 (0.31-2.15) | 0.674 |  |  |
