# Supplementary material for: Additional surgical procedures and perioperative morbidity in post-chemotherapy retroperitoneal lymph node dissection for metastatic testicular cancer in two intermediate volume hospitals
Source: World J Urol. 2020 May 5;39(3):839–46. doi: 10.1007/s00345-020-03229-5 (PMC7969692; doi:10.1007/s00345-020-03229-5)
Supplement: Supplementary file 2 — Supplementary file2 (DOCX 18 kb) [file 345_2020_3229_MOESM2_ESM.docx]

**Supplementary Table 2. Overview of types of complications**

| **Type of complications** | **Treatment** | **Overall** | **Template RPLND** | **RMR** |
| --- | --- | --- | --- | --- |
| **Clavien-Dindo Grade 2** |  | **24 (19.4)** | **12 (16.7)** | **12 (23.1)** |
| - Infection | Antibiotics | *14 (11.3)* | *8 (11.1)* | *6 (11.5)* |
| - Chylous leakage | Minimal chain diet | *3 (2.4)* | *2 (2.8)* | *1 (1.9)* |
| - Ileus | Enema / readmittance | *2 (1.6)* | *-* | *2 (3.8)* |
| - Anemia | Blood transfusion | *2 (1.6)* | *-* | *2 (3.8)* |
| - High blood pressure | Medication | *1 (0.8)* | *-* | *1 (1.9)* |
| - Pain | PCEA | *2 (1.6)* | *2 (2.8)* | *-* |
| **Clavien-Dindo Grade 3a** |  | **4 (3.2)** | **3 (4.2)** | **1 (1.9)** |
| - Chylous leakage | Percutaneous drainage | *2 (1.6)* | *1 (1.4)* | *1 (1.9)* |
| - Renal artery thrombosis | Thrombectomy | *1 (0.8)* | *1 (1.4)* | *-* |
| - Atrial fibrillation | Cardioversion | *1 (0.8)* | *1 (1.4)* | *-* |
| **Clavien-Dindo Grade 3b** |  | **5 (4.0)** | **4 (5.6)** | **1 (1.9)** |
| - Intestinal perforation / ischemia | Laparotomy | *2 (1.6)* | *1 (1.4)* | *1 (1.9)* |
| - Hydronephrosis | Double pigtail stent | *1 (0.8)* | *1 (1.4)* | *-* |
| - Metabolic instability, unknown cause | Laparotomy | *1 (0.8)* | *1 (1.4)* | *-* |
| - Compartment syndrome | Fasciotomy | *1 (0.8)* | *1 (1.4)* | *-* |
| **Clavien-Dindo Grade 4a** |  | **3 (2.4)** | **2 (2.8)** | **1 (1.9)** |
| - Tubular necrosis | Continuous hemofiltration, ICU | *1 (0.8)* | *1 (1.4)* | *-* |
| - Cardiac arrest | Resuscitation, ICU | *1 (0.8)* | *1 (1.4)* | *-* |
| - Retroperitoneal fluid collection with septic shock | Drainage, ICU | *1 (0.8)* | *-* | *1 (1.9)* |
| **Clavien-Dindo Grade 5** |  | **2 (1.6)** | **1 (1.4)** | **1 (1.9)** |
| - Postoperative bleeding | Surgery | *2 (1.6)* | *1 (1.4)* | *1 (1.9)* |

PCEA = Patient-controlled epidural analgesia; ICU = Intensive care unit; RMR = residual mass resection; RPLND = retroperitoneal lymph node dissection
