# Supplementary material for: Additional surgical procedures and perioperative morbidity in post-chemotherapy retroperitoneal lymph node dissection for metastatic testicular cancer in two intermediate volume hospitals
Source: World J Urol. 2020 May 5;39(3):839–46. doi: 10.1007/s00345-020-03229-5 (PMC7969692; doi:10.1007/s00345-020-03229-5)
Supplement: Supplementary file 3 — Supplementary file3 (DOCX 16 kb) [file 345_2020_3229_MOESM3_ESM.docx]

| **Supplementary Table 3. Predictors of a Complication Clavien-Dindo Grade ≥III** | | | | | | | |  |
| --- | --- | --- | --- | --- | --- | --- | --- | --- |
|  | **Univariate** | | | **Multivariate** | | | | |
|  | **OR (95% CI)** | **P-value** | | **OR (95% CI)** | | | **P-value** | |
| Age | 1.05 (0.99-1.10) | | 0.087 | |  |  | | |
| Retroperitoneal primary | 2.43 (0.59-10.06) | | 0.248 | |  |  | | |
| Seminoma primary | 1.04 (0.21-5.14) | | 0.958 | |  |  | | |
| IGCCCG intermediate / poor risk | 2.24 (0.66-7.57) | | 0.179 | |  |  | | |
| Tumor regression | 1.02 (0.99-1.05) | | 0.243 | |  |  | | |
| Residual tumor size >5 cm | 3.30 (0.97-11.17) | | 0.055 | | 2.26 (0.62-8.25) | 0.218 | | |
| Residual mass resection* | 1.31 (0.41-4.18) | | 0.640 | |  |  | | |
| Additional intervention | **4.48 (1.42-14.13)** | | **0.011** | | **3.46 (1.03-11.60)** | **0.044** | | |
| Histology RPLND specimen |  | | 0.520 | |  |  | | |
| - Necrosis / fibrosis | Reference | |  | |  |  | | |
| - Viable cancer | 0.80 (0.15-4.30) | |  | |  |  | | |
| - Teratoma | 0.50 (0.15-1.68) | |  | |  |  | | |

* Compared to template-based surgery

IGCCCG = International Germ Cell Cancer Group; OR = odds ratio; RMR = residual mass resection; RPLND = retroperitoneal lymph node dissection
